# Synergistic effects of rivaroxaban and hypothermia or acidosis on coagulation initiation measured with ROTEM^®^: a prospective observational study

**DOI:** 10.1186/s12959-024-00661-0

**Published:** 2024-10-18

**Authors:** Lotta Sunnersjö, Isak Ymén, Ulf Schött, Andreas Hillarp, Johan Undén, Thomas Kander

**Affiliations:** 1 Department of Intensive and Perioperative Care, Skåne University Hospital, Malmö, 214 28 Sweden; 2 The Medical Faculty, Lund University Sweden Sölveg, Lund, 223 62 Sweden; 3 Department of Intensive and Perioperative Care, Skåne University Hospital, Lund, 221 85 Sweden; 4 Department of Medical Biochemistry, Oslo University Hospital, Oslo, 0372 Norway; 5 Department of Translational Medicine, Skåne University Hospital, Lund University, Malmö, 205 02 Sweden; 6 Department of Intensive and Perioperative Care, Hallands Hospital, 302 33 Halmstad, Sweden

**Keywords:** Rivaroxaban, ROTEM, Hypothermia, Acidosis, Coagulation, Anticoagulant

## Abstract

**Background:**

Hypothermia and acidosis individually inhibit haemostasis. We designed this study with the aim to investigate whether rivaroxaban combined with hypothermia or acidosis exhibit synergistic inhibitory effects on haemostasis using ROTEM^®^.

**Methods:**

Patients with a clinical indication to start rivaroxaban treatment were prospectively included. Blood samples were collected before initiation of treatment and the day after. All blood samples were in vitro modified with respect to temperature (incubated and analysed at 28, 33, 37 and 40 degrees Celsius (°C)) and pH (6.8, 7.0, 7.2 and 7.4). The temperature and acidosis effects on the ROTEM EXTEM variables clotting time (CT), clot formation time (CFT) and alpha-angle (AA) were measured along with the individual effect of rivaroxaban on the same variables. The additive effect was calculated. The observed (potential synergistic) effects for the temperature and pH modified rivaroxaban samples on the same ROTEM variables, were registered. Differences between the additive and observed (potential synergistic) effects were analysed using matched non-parametric hypothesis testing.

**Results:**

In total, 13 patients were included. Hypothermia and rivaroxaban exhibited a synergistic effect on CT at 28 °C (*p* = 0.0002) and at 33 °C (*p* = 0.0007). The same applied for acidosis at pH 6.8 (*p* = 0.003) and pH 7.0 (*p* = 0.003). There were no signs of synergistic effects of rivaroxaban and temperature or acidosis on CFT. In AA there were signs of synergism at 28 °C (*p* = 0,001), but not at other tested temperatures or pH levels.

**Conclusions:**

The combination rivaroxaban together with hypothermia or acidosis demonstrated inhibitory synergistic effects on haemostasis.

**Trial registration:**

The study was retrospectively registered 2023-03-01 at ClinTrials.gov with NCT05669313.

**Supplementary Information:**

The online version contains supplementary material available at 10.1186/s12959-024-00661-0.

## Background

After the introduction of direct oral anticoagulants (DOACs) the number of people in the world using these medications has almost doubled in the last decade [1, 2]. Considering the side effect of increased risk of bleeding, the management of haemorrhage and coagulopathy following clinical situations in these patients, such as trauma, is vital [3, 4].

Previous studies have demonstrated that hypothermia weakens the secondary haemostasis due to reduction of fibrinogen synthesis and progressive inhibition of the initiation phase of thrombin generation [5–8]. In trauma patients, temperatures below 34 degrees Celsius (°C) were identified as a threshold beneath which significant coagulopathy appears [9]. For the intra-operative setting, a meta-analysis of 14 randomised controlled studies demonstrated that even mild hypothermia (< 1 ֯C) significantly increased blood loss [10].

Acidosis, however, does not seem to affect the initiation phase of thrombin generation, but rather the propagation phase. Decreasing levels of pH have been linked to impaired clot formation and in animal models the induction of acidosis resulted in increased fibrinolysis and caused coagulation dysfunction that persisted even after pH was normalised [7].

Rotational thromboelastometry (ROTEM) is a point-of-care viscoelastic method widely used in the management of bleeding caused by trauma and during cardiothoracic surgery, linked with a decreased need for blood products and rapid identification of coagulopathy [11, 12]. The effects of hypothermia and acidosis on whole blood coagulation in vitro have previously been studied using ROTEM [5]. For hypothermia, a significant increase in clotting time (CT) and clot formation time (CFT) was observed, while acidosis alone did not alter ROTEM indices.

The ROTEM assay EXTEM has previously been shown to be more sensitive to elevated concentrations of DOACs in blood compared with other ROTEM assays, with a demonstrated linear relationship between increasing concentrations of DOACs and prolonged CT [13, 14]. However, to the best of our knowledge, the possible synergistic effects between DOACs and acidosis or hypothermia on the EXTEM-assay have not been explored. Given the increased prevalence of patients treated with DOACs and that point of care thromboelastometry is increasingly common to guide transfusion therapy in critical bleeding, we designed the current experimental study with the aim of investigating the effects of rivaroxaban together with hypothermia or acidosis using the ROTEM assay EXTEM. The EXTEM assay together with the FIBTEM assay are the most commonly used ROTEM assays in trauma and other critical bleeding situations [15]. The primary aim of this study was to investigate possible synergistic effects between rivaroxaban and hypothermia or acidosis, detected in the CT of the ROTEM EXTEM-assay. The secondary aims were to investigate the same effects on CFT and alpha-angle (AA) of hypothermia and acidosis in the same assay. The hypothesis was that synergistic effects of rivaroxaban and hypothermia or acidosis could be identified.

## Methods

This prospective observational study was approved by the Swedish Ethical Review Authority (Dnr: 2022-02275-01) and all participants gave written informed consent prior to inclusion. The manuscript was written in accordance with the STROBE guidelines [16].

Patients over 18 years of age, reporting to the emergency department during office hours at Skåne University Hospital Lund, Sweden, from September to November 2022, were recruited for the study. The inclusion criterion was any indication for starting treatment with rivaroxaban. Exclusion criteria were previously diagnosed coagulopathy or medication within the last month with anticoagulants including vitamin K antagonists (VKAs) and DOACs, platelet inhibitors (not acetylsalicylic acid) and non-steroidal anti-inflammatory drugs.

### Sample collection and baseline characteristics

Baseline blood samples were taken prior to the first peroral dose of rivaroxaban (Xarelto^®^, Bayer AG, Leverkusen, Germany;15 mg) and patients were instructed to take another dose of rivaroxaban, 15 mg, the following morning. This is in line with recommendations for treatment of acute onset venous thromboembolism. At their arrival the following morning, they were asked for compliance with the rivaroxaban medication and were excluded if rivaroxaban had not been taken as prescribed. At the follow up visit, a second set of blood samples was taken, with the aim of reaching the maximum plasma concentration of rivaroxaban in the blood. A blood sample for analysis of rivaroxaban concentration was also taken. On both occasions venous blood was drawn in 2.7 ml tubes containing 0.109 M citrate (BD Vacutainer Systems, Plymouth, UK) from an antecubital vein. Baseline characteristics and results from routine laboratory analyses were manually collected from medical records.

### In vitro modification

Immediately after blood sampling, 1 ml of blood was transferred to each of eight different plastic test tubes (Sarstedt, Micro tube 1.5 ml, Sarstedt AG & Co. KG, Nümbrecht, Germany) and divided into one temperature group and one acidosis group, each consisting of four vials.

To ensure the detectability of any hypothermic effect, even in cases where it may be subtle, we opted for temperatures ranging from 28 to 40 degrees Celsius (°C). The temperature group vials were incubated in water baths for 20 min at their target goal temperatures of 28, 33, 37, and 40 °C. Prior to utilizing the ROTEM devices, the temperature within the ROTEM system was adjusted to the specified testing temperature, allowing sufficient time for proper equilibration. The acidosis group was incubated at 37 °C and mixed with 15 µL of hydrochloric acid (HCl) at concentrations of 0.15 µM, 0.10 µM, 0.05 µM or sterile water, compensating for any dilutional effects from the HCl. Titration with the different concentrations of HCl was tested prior to the current study using blood from healthy volunteers, with the goal of reaching the clinically relevant, pre-defined pH levels of 6.80, 7.00, and 7.20. After the addition of hydrochloric acid and sterile water the pH in each tube was measured using a pH-meter (Testo, Testo 230 pH/Temperature Meter, Testo SE & Co. KGaA, Lenzkirch, Germany). All ROTEM assays were performed within four hours of drawing blood.

### ROTEM

After incubation at different temperatures and the addition of HCl/sterile water, EXTEM assays were analysed using newly served, calibrated and tested ROTEM delta (ROTEM, ROTEM delta, TEM innovations, Munich, Germany) or Rotational thromboelastography (roTEG 05, TEM innovations, Munich, Germany). All assays were performed according to the manufacturer’s instructions. Due to time restrictions (4 h after sampling) we chose to use both ROTEM delta and roTEG. CT, AA, and CFT were registered. Maximal clot firmness (MCF) was not registered as previous studies with in-vitro modification to hypothermia were without effect on MCF [7]. All tests were run with the ROTEM instrument set to the incubation temperature.

### Statistical analysis

We wanted to demonstrate a 25% higher synergistic effect of rivaroxaban and hypothermia at 28 °C in the ROTEM EXTEM CT assay compared to the calculated additive effect (see below) with a non-parametric, matched hypothesis test. Given a standard deviation of 19, a 90% power and an alpha-error probability of 0.01 we had to include 13 patients. To allow for dropouts we aimed to include 15 patients.

All continuous variables are presented as medians and ranges (min-max). All numbers are presented as percentages (%).

The temperature and acidosis groups both at baseline and after exposure to rivaroxaban were compared within themselves using the non-parametric Friedman test to evaluate the observed effects of hypothermia, acidosis, hypothermia with rivaroxaban, and acidosis with rivaroxaban on the ROTEM variables CT, AA and CFT. If the Friedman statistics were significant, post hoc Dunn’s multiple comparisons test was performed to investigate differences between different levels of temperature and pH compared to baseline (37 °C and pH 7.4).

To test for possible synergy between rivaroxaban and hypothermia or rivaroxaban and acidosis, a theoretical additive effect was calculated as a sum of the pure temperature or acidosis effect and the pure rivaroxaban effect. The pure temperature or pure acidosis effect was the difference in a variable (CT, CFT or AA) from baseline, where baseline was the value of that variable at 37 °C and pH 7.4. The rivaroxaban effect was calculated by subtracting the value of a rivaroxaban test (CT, CFT or AA) at a specific temperature or pH with the value at baseline defined as the rivaroxaban test at 37 °C and pH 7.4 respectively. This additive effect was then compared to the real observed effect of simultaneous hypothermia/acidosis and rivaroxaban. Hence, if there is no synergy between rivaroxaban or acidosis/hypothermia, there would be no difference between additive and observed effects. If synergy exists however, the observed effect would systematically be greater than the additive effect. After calculating the values of the additive and observed effects, they were compared using the two-tailed, non-parametric Wilcoxon matched-pairs signed rank test. The Wilcoxon test was also used to test possible pH differences between baseline and rivaroxaban samples.

A possible dose-response relationship of the synergy between rivaroxaban and hypothermia or acidosis on ROTEM variables was also investigated. This was only done if a significant synergy could be demonstrated in the pairwise testing of differences between additive and synergistic effects. To verify normally distributed residuals, four tests of normality were used: the D’Agostino-Pearson omnibus test, the Anderson-Darling test, the Shapiro-Wilk test, and the Kolmogorov-Smirnov test. To test if differences between the slopes of the lines, indicating dose-response synergy, a null-hypothesis of parallel lines was assumed generating a *p*-value to answer the question as to what the probability of the observed slopes is provided that the null hypothesis holds. Analyses for the study were performed using GraphPad Prism version 9.5.0 (GraphPad, San Diego, USA). Due to the large number of statistical tests planned a modified Bonferroni correction was established placing a requirement of *p* < 0.01 for statistical significance.

## Results

**Fig. 1:**
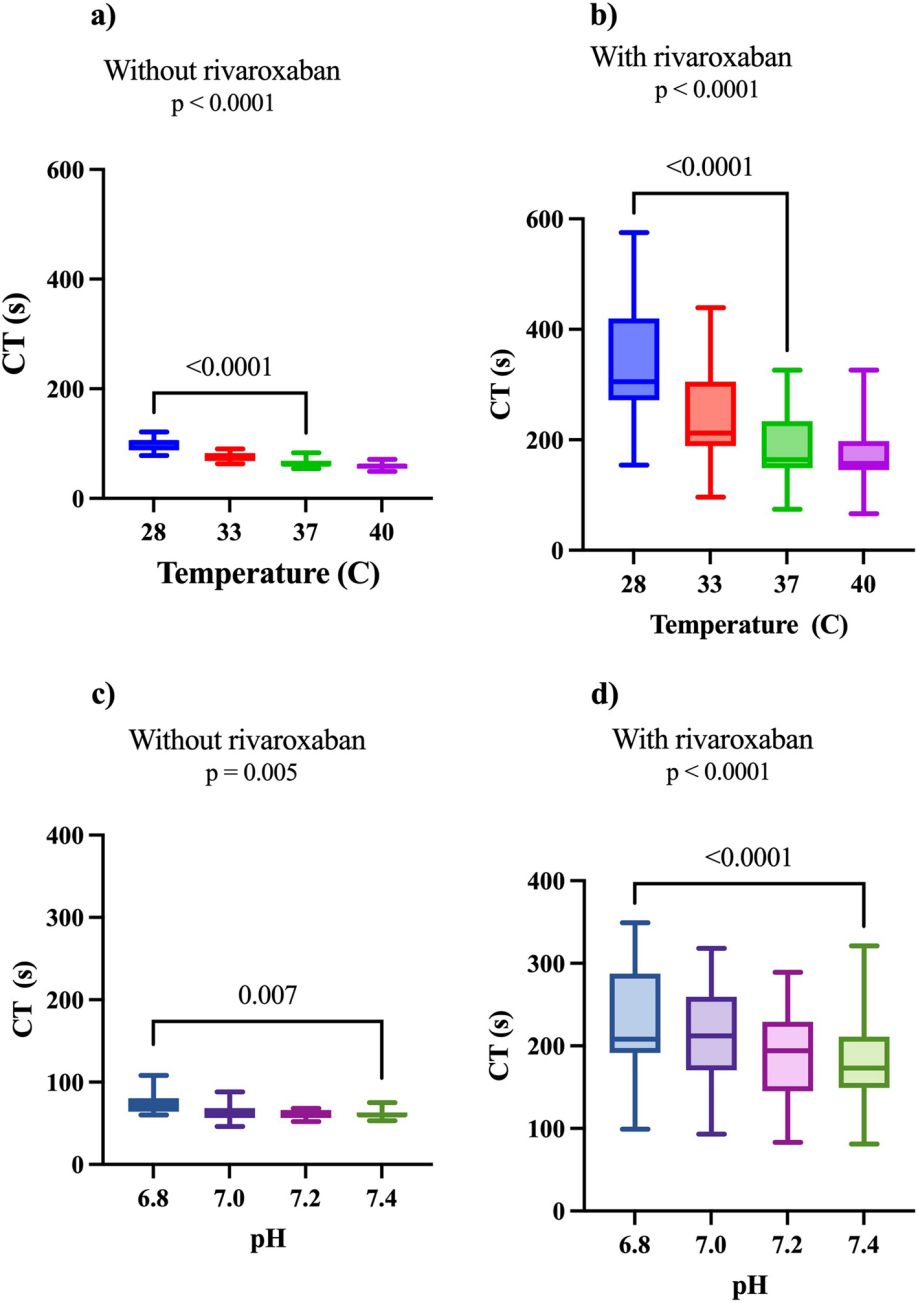
Comparison of clotting time with and without rivaroxaban at modified temperatures and pH. Clotting time (CT) with in-vitro modifications of temperatures and pH. *P*-value for Friedmans test. Significant *p*-values for Dunn’s multiple comparisons are displayed. Whiskers represent the minimum to maximum range

In total 15 patients under novel investigation/treatment for deep vein thrombosis were included. Two patients were excluded because they did not take the second dose of rivaroxaban, leaving 13 patients who formed the study cohort. The baseline characteristics of included patients are presented in Table 1. Details on the effects of hypothermia and acidosis with and without rivaroxaban on CT are displayed in Fig. 1. In summary, CT-values were different both with and without rivaroxaban after modification of both temperature and pH.

**Table 1:** Baseline characteristic^a^

	*n* = 13 (100)
Age	66 (32–89)
Men	7 (54)
Diabetes mellitus 1 or 2	2 (15)
Cardiovascular disease	2 (15)
Kidney disease	1 (8)
Hypertension	6 (46)
Previous VTE^b^	5 (38)
Cancer	1 (8)
Haemoglobin (g/L)	141 (115–157)
PT (INR)^c^	0.95 (0.8–1.2)
APTT (s)^d^	25 (23–30)
Platelet count (x10^9^/L)	299.5 (156–350)
Creatinine (µmol/L)	80 (53–166)
eGFR (µmol/L)^e^	78 (22–105)

^a^Numbers are presented with percentages and continuous variables are presented with the median (min-max)

^b^Venous thromboembolism

^c^Prothrombin time (international normalised ratio)

^d^Activated partial thromboplastin time

^e^Estimated glomerular filtration rate

**Fig. 2:**
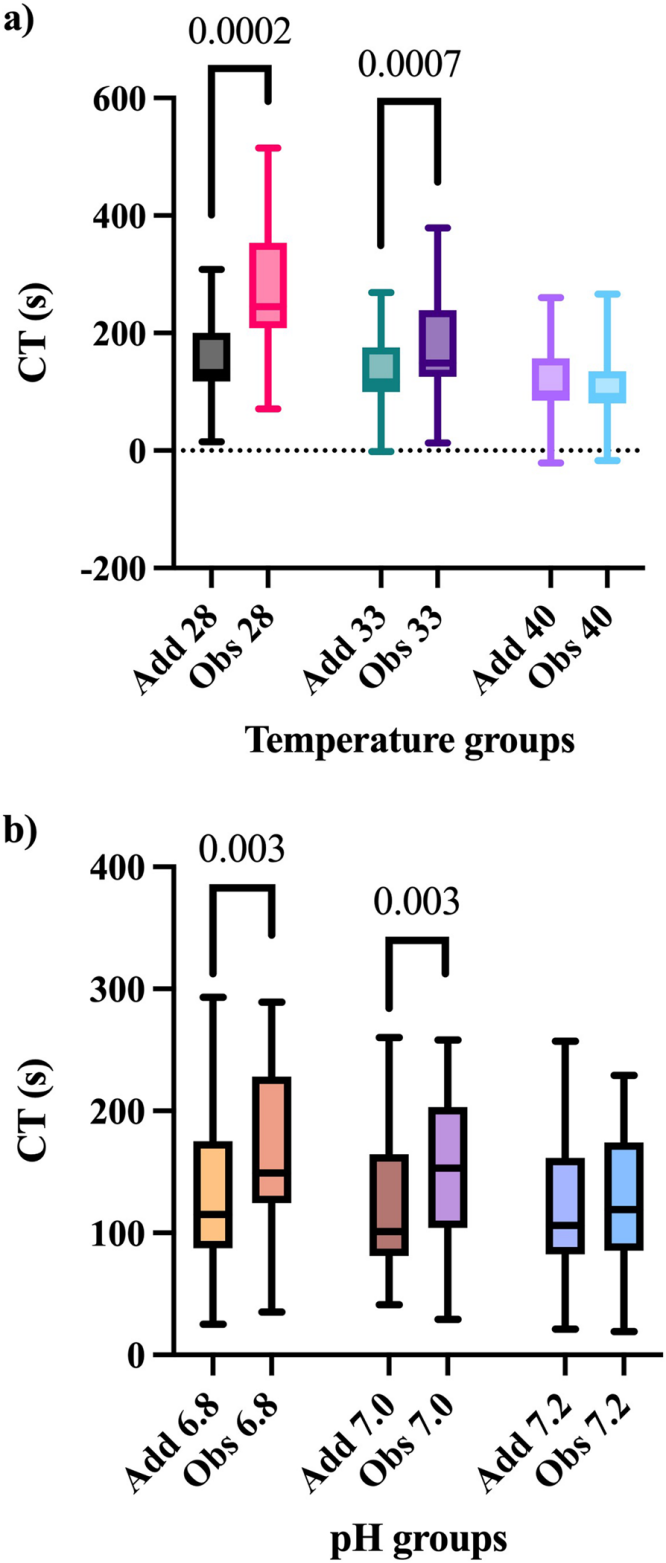
Comparisons between additive effect and observed (potential synergistic) effect on clotting time. Comparisons between additive effects and observed (potential synergistic) effects on clotting time.Significant *p*-values for pairwise comparisons with the Wilcoxon signed rank test are displayed. Add = calculated additive effect of temperature and rivaroxaban. Obs = observed effect of rivaroxaban and temperature or acidosis (synergistic effect if significantly higher than the additive effect). Whiskers represent minimum to maximum range

At 28 °C the median additive effect was 133 s (15;308) and the median observed effect was 245 s (71;515), *p* = 0.0002. At 33 °C the median additive effect was 117 s (-2;269) and the median observed effect was 149 s (13;379), *p* = 0.0007 as shown in Fig. 2. This indicates a synergistic CT prolongation of rivaroxaban and hypothermia to 28 and 33 °C. The same was true at pH 6.8 with a median additive effect of 115 s (25;293) and a median observed effect of 149 s (35;289), *p* = 0.003. At pH 7.0 the median additive effect was 101 s (41;260) and the median observed effect was 153 s (29;258), *p* = 0.003, displaying a synergistic effect on CT. Neither the 40-degree comparison nor the pH 7.2 comparison demonstrated statistically significant results.

**Fig. 3:**
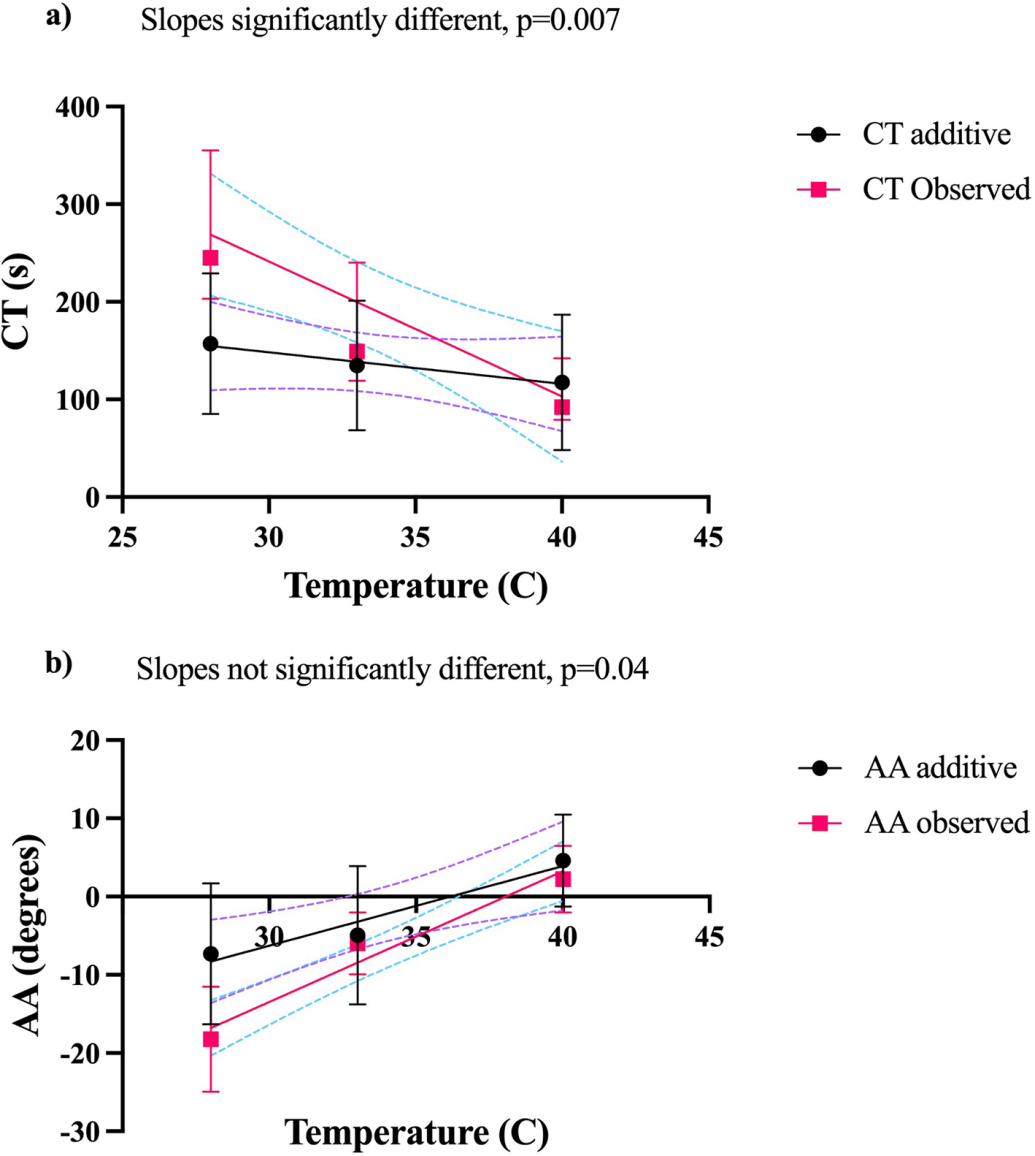
Linear regression of additive and observed (potential synergistic) effects on clotting time and alpha-angle at different temperatures. Linear regression with 95% confidence interval (dashed line) of additive and observed (potential synergistic) effects on clotting time and alpha-angle at different temperatures. Whiskers represent standard deviations. a) represents CT, b) represents AA. Details on the effects of hypothermia and acidosis with and without rivaroxaban on CFT and AA are displayed in Figs. 4 and 5. In summary, CFT- and AA-values were different both with and without rivaroxaban after modification of temperature and pH with the exception of AA without rivaroxaban

 For hypothermia, the residuals of CT of both the additive effect and observed effect passed all four tests of normality. Hence, simple linear regression was allowed, showing a significant difference in slope between the observed and additive groups (*p* = 0.007) (Fig. 3). For acidosis the residuals of the additive and observed effects of CT did not pass the normality tests or after logarithmization, hence, linear regression could not be performed.

**Fig. 4:**
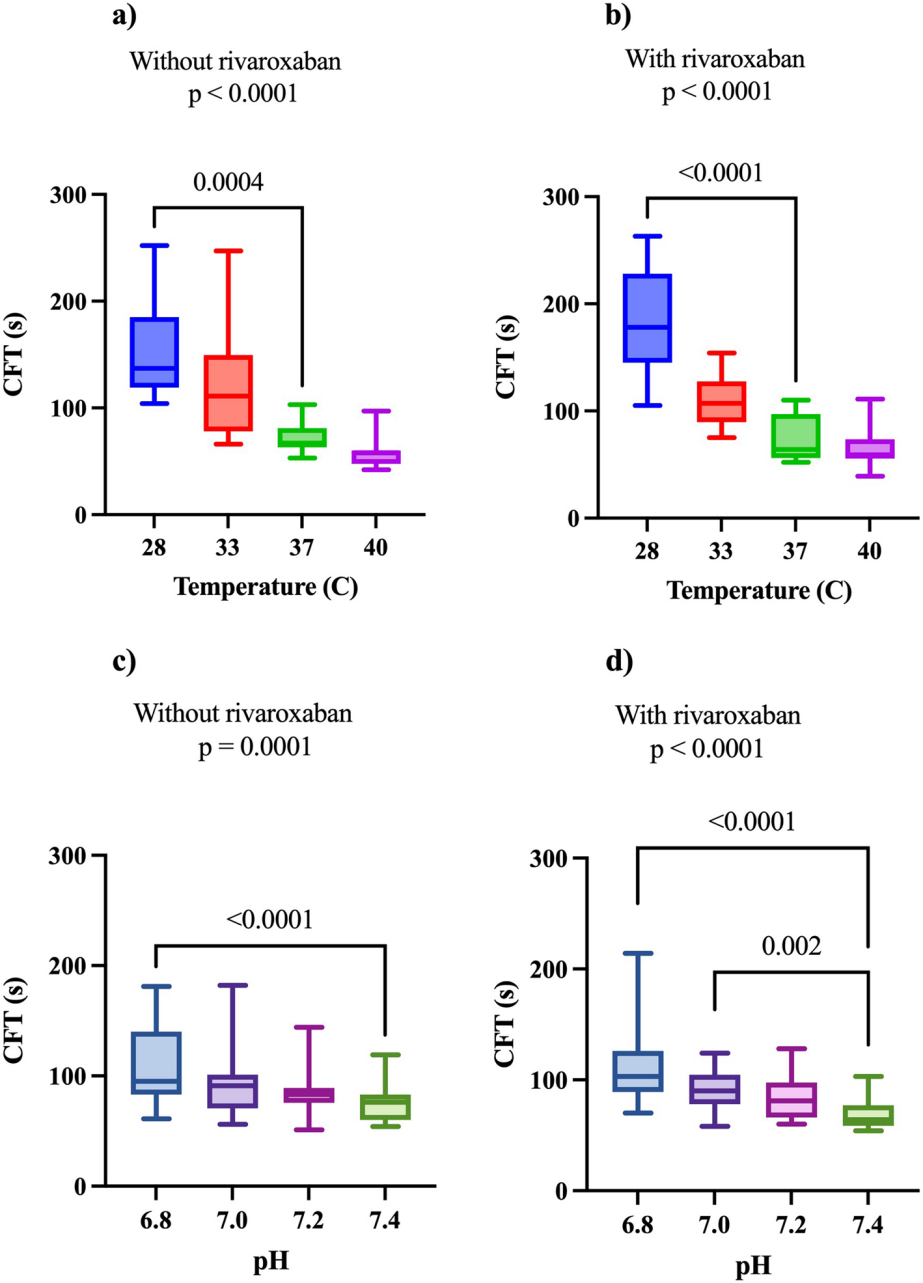
Comparison of clot formation time with and without rivaroxaban at modified temperatures and pH. Comparison of clot formation time (CFT) with and without rivaroxaban at modified temperatures and pH. *P*-value for Friedmans test. Significant *p*-values for Dunn’s multiple comparisons are displayed. Whiskers represent the minimum to maximum range

**Fig. 5:**
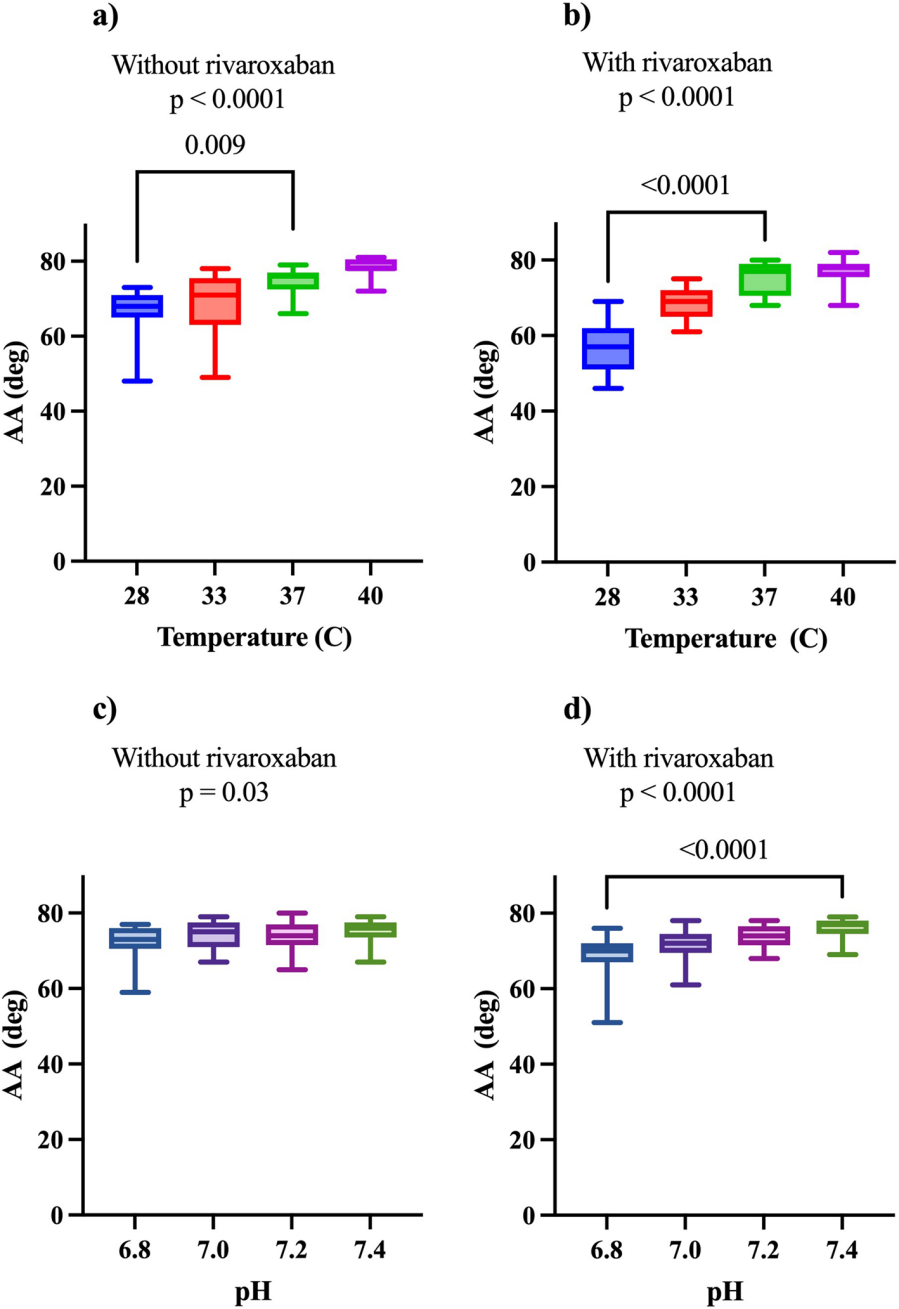
Comparison of alpha-angle with and without rivaroxaban at modified temperatures and pH. Comparison of alpha-angle (AA) with and without rivaroxaban at modified temperatures and pH. *P*-values for Friedmans test. Significant *p*-values for Dunn’s multiple comparisons are displayed. Whiskers represent the minimum to maximum range

There were no signs of synergistic effects of rivaroxaban and hypothermia or acidosis on CFT, (Additional file 1).

There were signs of synergistic effects on AA of rivaroxaban and hypothermia at 28 °C but not at any other tested temperature or pH (Additional file 2).

Linear regression could not be performed for additive and observed temperature effects of CFT as the residuals did not pass the normality testing nor after logarithmization. For AA the slopes of the lines representing additive and observed (potential synergistic) did not differ. (Fig. 3b).

The median rivaroxaban concentration in the study population was 316 µg/L (53–531 µg/L). Pairwise comparisons of the level of pH in baseline samples compared with the pH in matching rivaroxaban samples showed no significant differences between any of the pH groups (Additional file 3).

The median actual pH-values in the samples were 6,73 (6,57 − 6,81) in the 6.8-group, 6,93 (6,79 − 7,01) in the 7.0-group and 7,16 (7,04–7,23) in the 7.2 group.

## Discussion

In this prospective observational study, we demonstrated that rivaroxaban in combination with hypothermia significantly increased CT more than what could be explained by their separate effects added together, thus confirming the hypothesis that synergistic effects of rivaroxaban and hypothermia could be detected in the CT of the ROTEM EXTEM-assay. Linear regression demonstrated a steeper slope for the observed effect of rivaroxaban with hypothermia on CT compared with their additive effects, suggesting a synergistic dose-response between rivaroxaban and hypothermia. Together with acidosis, rivaroxaban caused a significant increase in CT compared to the expected additive effect, thus also suggesting synergy between rivaroxaban and acidosis. To the best of our knowledge, this synergy between rivaroxaban and hypothermia or acidosis has never previously been demonstrated.

Our results indicate that hypothermia and rivaroxaban do not seem to confer a synergistic effect on CFT as no significant differences could be detected between the additive and observed effects of CFT (Additional file 1). For AA a synergistic effect of hypothermia at 28 °C together with rivaroxaban could be detected (Additional file 2). However, this synergistic effect was not present at 33 °C and there was no difference in slopes between additive and observed (potential synergistic) effects of hypothermia and rivaroxaban (Fig. 3b), indicating the absence of synergism on AA.

We could also show that hypothermia alone increased both CT and CFT and reduced AA. These findings are in line with several previous studies [5, 7, 17].

Acidosis prolonged CT and CFT but did not by itself reduce AA significantly. Previous studies show varying results after in-vitro acidification where some results demonstrate prolongation in CFT, a reduction of AA, and no alteration of CT [18, 19] while others show no effect on any ROTEM variable after acidosis pH ≥ 7.0 [5]. Likely, many explanations for these varying results can be found where one is different laboratory techniques. To achieve the lowest possible dilution in modified samples, we performed a pre-study where optimal HCl-concentrations and volumes were titrated. We noted that high molarity HCl gave pronounced haemolysis and diluted HCl did not give the desired effect. As described in the Methods sect, we identified 15 µL of HCl at concentrations from 0.05 to 0.15 µM to be ideal for the current experiment.

A strength of the current study is that the participants included were their own controls, hence avoiding many interpersonal variations as confounding factors. For patients experiencing acute onset venous thromboembolism, the recommended dosage for the initial three weeks consists of 15 mg twice daily to ensure prompt and effective treatment. This is then followed by a maintenance dosage of 20 mg daily. In our study, despite administering only two doses of 15 mg each, all rivaroxaban concentrations remained within the expected range for the subsequent daily dosage of 20 mg [20]. This makes the results clinically relevant for patients with a standard dosage of 20 mg daily and underline the importance of prompt correction of hypothermia and acidosis in bleeding patients on rivaroxaban.

The demonstrated synergies of rivaroxaban and hypothermia or acidosis on coagulation suggest that rapid administration of therapeutics directed towards restoration of secondary haemostasis could be beneficial for patients suffering from bleeding and hypothermia or acidosis. These effects do not however encompass the full spectrum of complexity involved in major bleeding and different diagnostic measurements and treatments should be used for the detection and correction of other defects in the secondary haemostasis and fibrinolysis, along with optimisation of the primary haemostasis [21, 22].

We recognise several limitations. Firstly, as a prospective observational study recruitment of patients, the study did not contain any elements of randomisation, and hence allowed for possible selection bias. Second, to minimise the blood volume collected from patients, blood was transferred from test tubes to 1.5 ml plastic cups. This increased the exposure of sample blood to air, allowing for diffusion of carbon dioxide potentially alkalising the blood and affecting the bicarbonate buffer system [23]. Third, we carefully titrated the minimal volume and concentration of the HCL that needed to be added and did not observe any signs of haemolysis. However, we did not measure the concentration of free haemoglobin, which has the potential to affect the results [24]. Fourth, we used two different instruments for thromboelastometry from the same manufacturer (ROTEM delta and roTEG), which could affect the results. However, in a recent study we have demonstrated the two instruments in our laboratory to be interchangeable [25]. Fifth, we did observe a small variation in pH levels in samples after titration with equal amounts of HCl. However, since the pairwise comparison between matched pH groups was not significant, there was no systematic difference in pH that affected the final results of the study. Sixth, we only examined the ROTEM EXTEM assay, which has shown the strongest correlation with DOAC [13, 14]. Further studies are needed to explore the effects on the other ROTEM assays. Seventh, the lowest temperatures tested are rare in clinical practice which warrants further studies that focus on mild hypothermia. Eighth, Although, the median concentration of rivaroxaban in the current study corresponds to the normal reference range of rivaroxaban in steady state, the current blood samples were taken after.

## Conclusions

In this prospective experimental study, we demonstrated synergistic effects of rivaroxaban with hypothermia and acidosis on the CT of the ROTEM EXTEM-assay and thus confirmed the hypothesis of the study. This was also confirmed for AA but not for CFT. As prolonged CT is an indicator of severe coagulation factor deficiency, clinicians should act promptly to correct hypothermia and acidosis in bleeding patients on rivaroxaban.

## Supplementary Information

Supplementary Material 1.

Supplementary Material 2.

Supplementary Material 3.

## Abbreviations

DOAC: Direct oral anticoagulant
VKA: Vitamin K antagonist
ROTEM: Rotational thromboelastometry
roTEG: Rotational thromboelastography
CT: Clotting time
CFT: Clot formation time
AA: Alpha-angle
MCF: Maximum clot firmness
HCl: Hydrochloric acid
