# Supplementary material for: Synergistic effects of rivaroxaban and hypothermia or acidosis on coagulation initiation measured with ROTEM®: a prospective observational study
Source: Thromb J. 2024 Oct 18;22:91. doi: 10.1186/s12959-024-00661-0 (PMC11488277; doi:10.1186/s12959-024-00661-0)
Supplement: Supplementary file 1 — Supplementary Material 1. [file 12959_2024_661_MOESM1_ESM.docx]

**Additional file 1. Comparison between additive and observed (potential synergistic) effect on clot formation time**


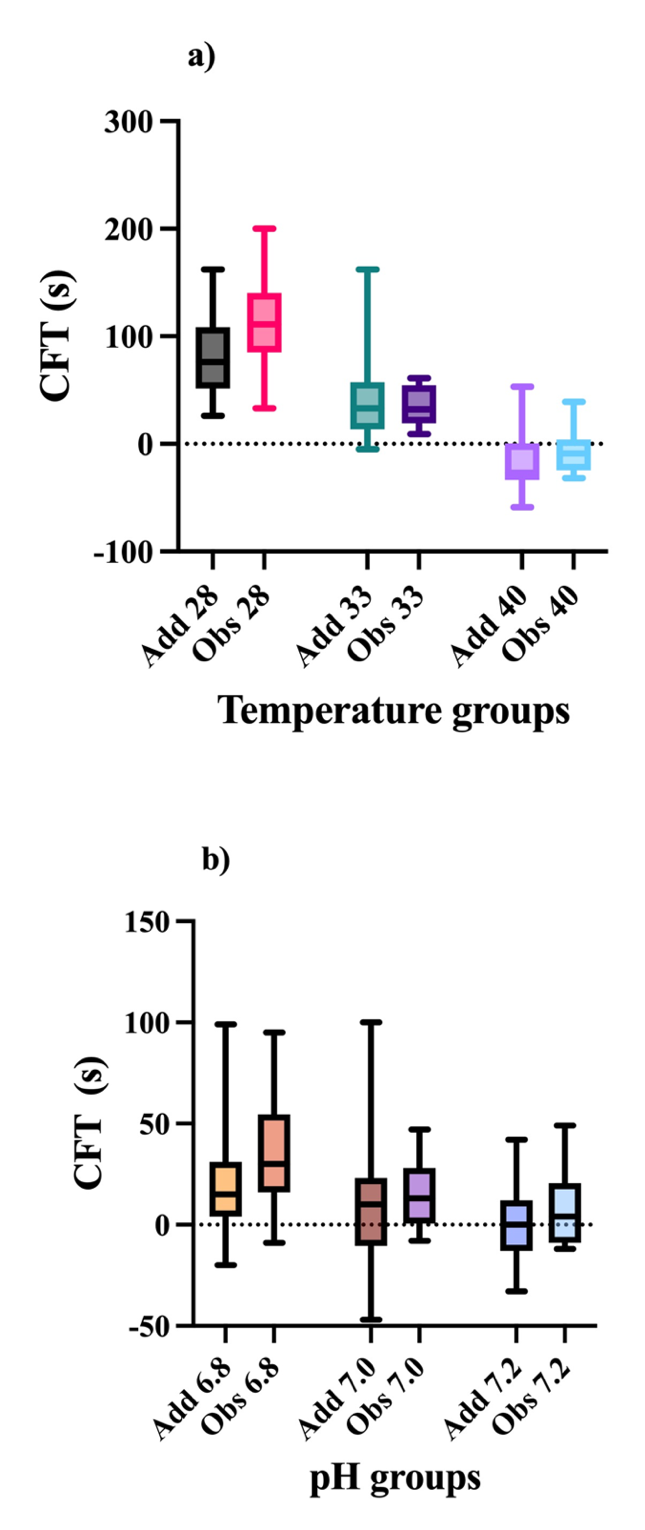


**Additional file 1**. Comparison between additive and observed (potential synergistic) effect on clot formation time. Significant P-values for pairwise comparisons with the Wilcoxon paired rank sum test are displayed in above boxplots. Add=calculated additive effect of temperature and rivaroxaban. Obs=observed effect of rivaroxaban and temperature or acidosis (synergistic effect if significantly higher than the additive effect). Whiskers represent minimum to maximum range.
