# Supplementary material for: Synergistic effects of rivaroxaban and hypothermia or acidosis on coagulation initiation measured with ROTEM®: a prospective observational study
Source: Thromb J. 2024 Oct 18;22:91. doi: 10.1186/s12959-024-00661-0 (PMC11488277; doi:10.1186/s12959-024-00661-0)
Supplement: Supplementary file 2 — Supplementary Material 2. [file 12959_2024_661_MOESM2_ESM.docx]

**Additional file 2. Comparison between additive and observed (potential synergistic) effect on alpha-angle**

**
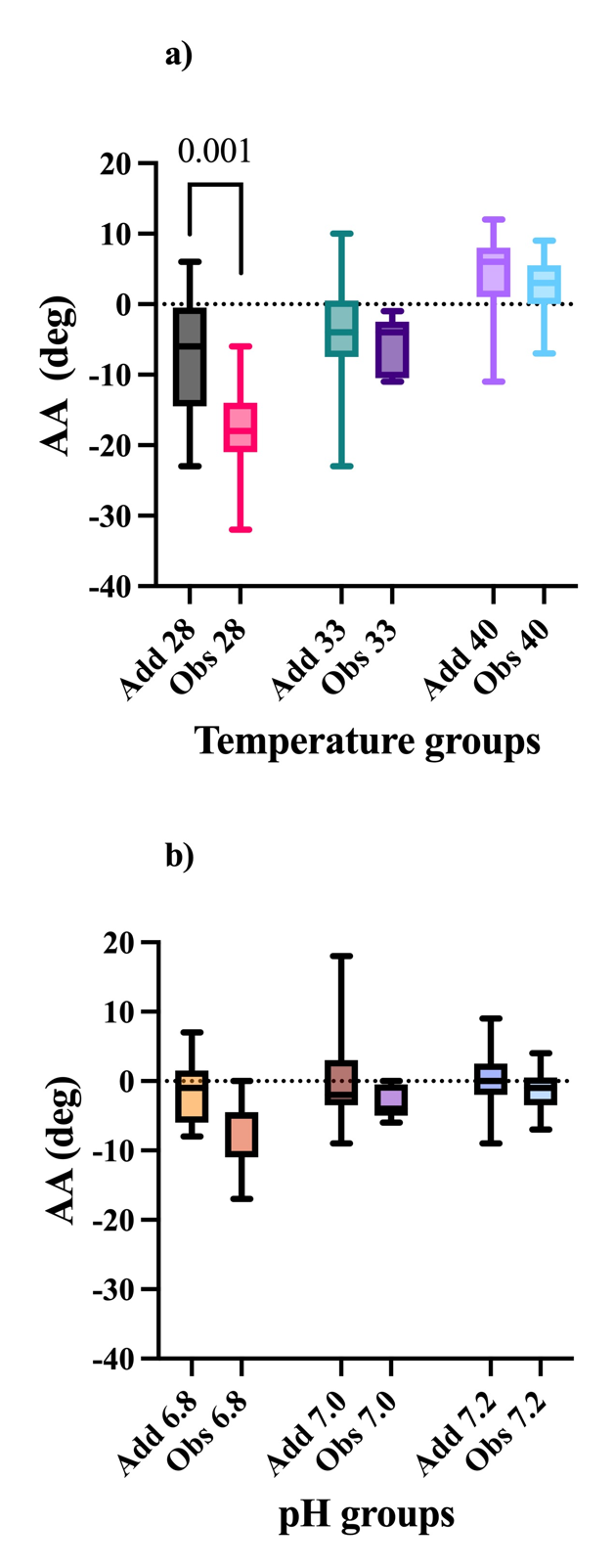
**

**Additional file 2.** Comparison between additive and observed (potential synergistic) effect on alpha-angle. Significant P-values for pairwise comparisons with the Wilcoxon paired rank sum test are displayed above boxplots. Add=calculated additive effect of temperature and rivaroxaban. Obs= observed effect of rivaroxaban and temperature or acidosis (synergistic effect if significantly higher than the additive effect). Whiskers represent minimum to maximum range.
