# Supplementary material for: Synergistic effects of rivaroxaban and hypothermia or acidosis on coagulation initiation measured with ROTEM®: a prospective observational study
Source: Thromb J. 2024 Oct 18;22:91. doi: 10.1186/s12959-024-00661-0 (PMC11488277; doi:10.1186/s12959-024-00661-0)
Supplement: Supplementary file 3 — Supplementary Material 3. [file 12959_2024_661_MOESM3_ESM.docx]

**Additional file 3. Comparison of pH between samples with and without Rivaroxaban**


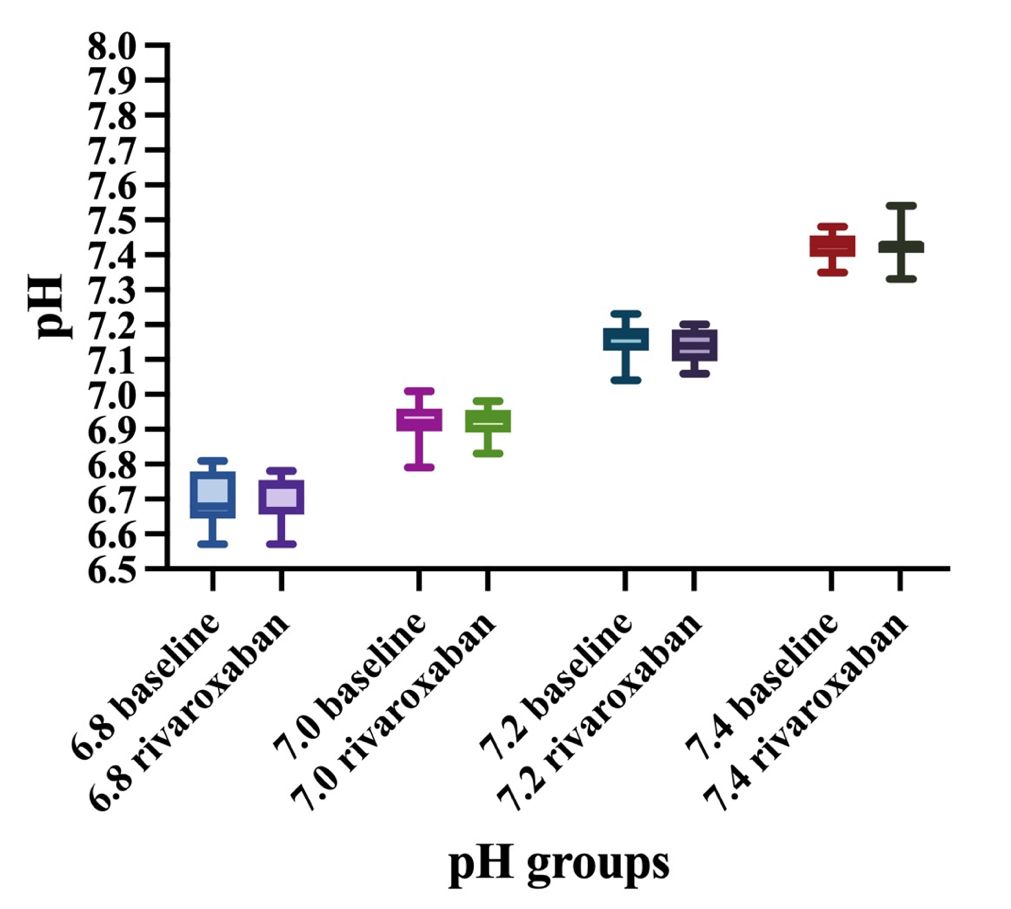


**Additional file 3.** Comparison of pH between samples with and without Rivaroxaban. Wilcoxon matched pairs-signed rank test is displayed above in boxplots. Whiskers represent minimum to maximum range.
